# Low-Level Lead Exposure, Metabolic Syndrome, and Heart Rate Variability: The VA Normative Aging Study

**DOI:** 10.1289/ehp.8992

**Published:** 2006-08-03

**Authors:** Sung Kyun Park, Joel Schwartz, Marc Weisskopf, David Sparrow, Pantel S. Vokonas, Robert O. Wright, Brent Coull, Huiling Nie, Howard Hu

**Affiliations:** 1 Department of Environmental Health, Harvard School of Public Health, Boston, Massachusetts, USA; 2 Channing Laboratory, Department of Medicine, Brigham and Women’s Hospital, Harvard Medical School, Boston, Massachusetts, USA; 3 VA Normative Aging Study, Veterans Affairs Boston Healthcare System and the Department of Medicine, Boston University School of Medicine, Boston, Massachusetts, USA; 4 Department of Biostatistics, Harvard School of Public Health, Boston, Massachusetts, USA

**Keywords:** autonomic nervous system, bone lead, heart rate variability, hypertension, metabolic syndrome

## Abstract

**Background:**

Altered heart rate variability (HRV), a marker of poor cardiac autonomic function, has been associated with sudden cardiac death and heart failure.

**Objective:**

We examined the association of low-level lead exposure measured in bone by K-X-ray fluorescence with alterations in HRV, and whether metabolic syndrome (MetS) or its individual components modify those associations.

**Methods:**

HRV measures [power in high-frequency (HF_norm_) and low-frequency (LF_norm_) in normalized units, and LF/HF] were taken among 413 elderly men from the Normative Aging Study. MetS was defined as subjects having three or more of the following criteria: abdominal obesity, hypertriglyceridemia, low high-density lipoprotein, high blood pressure, and high fasting glucose.

**Results:**

Of the subjects, 32% were identified as having MetS. Inverse but nonstatistically significant associations of both tibia and patella lead levels with HF_norm_ and nonstatistically significant positive relations with LF_norm_ and LF/HF were found in the entire cohort. There was a graded, statistically significant reduction in HF_norm_ and increases in LF_norm_ and LF/HF in association with an increase in patella lead as the number of metabolic abnormalities increased. We also observed that higher patella lead was consistently associated with lower HF_norm_ and higher LF_norm_ and LF/HF among subjects with MetS or its individual components. No statistically significant interaction between MetS and tibia lead was observed.

**Conclusion:**

The results suggest that elderly men with MetS were more susceptible to autonomic dysfunction in association with chronic lead exposure as measured in patella. The modification by MetS is consistent with a role for oxidative stress in lead toxicity on the cardiovascular system.

Exposure to lead is known to affect the cardiovascular system, even at low, general environmental levels. Many epidemiologic studies have shown an association between chronic low-level lead exposure and hypertension (Hu et al. 1996; Korrick et al. 1999; Nash et al. 2003) and cardiovascular disease (Cheng et al. 1998; Lustberg and Silbergeld 2002; Schwartz 1991). One possible mechanism for this association is interference in autonomic nerve control of the heart. Experimental studies have shown that lead can generate reactive oxygen species (ROS) by depletion of glutathione and protein-bound sulfhydryl groups, leading to oxidative stress (Gurer and Ercal 2000). Oxidative stress plays an important role in the production of proinflammatory mediators, lipid peroxidation, the suppression of nitric oxide, and alteration of calcium homeostasis, which may increase central sympathetic nerve activity and reduce baroreflex sensitivity and vagal parasympathetic tone (Ding et al. 2000; Dursun et al. 2005; Vaziri 2002).

Heart rate variability (HRV) is a non-invasive and quantitative marker of cardiac autonomic function that reflects the regulation of the sinoatrial node by the sympathetic and parasympathetic branches of the autonomic nervous system [Task Force of the European Society of Cardiology and the North American Society of Pacing and Electrophysiology (Task Force) 1996]. Decreased HRV is an independent predictor of mortality in middle-aged and elderly subjects, in patients with diabetes, and in survivors of myocardial infarction and other coronary heart diseases (Gerritsen et al. 2001; Tapanainen et al. 2002; Tsuji et al. 1996). Several studies of workers occupationally exposed to lead support the contention that lead acts upon the heart via the autonomic nervous system (Bockelmann et al. 2002; Murata and Araki 1991; Murata et al. 1993; Teruya et al. 1991). A recent study from Korea on subjects nonoccupationally exposed to lead showed significant univariate associations between blood lead and HRV measures, but failed to find the associations after adjustment for confounding variables, such as age, alcohol consumption, and other blood metal concentrations (Jhun et al. 2005). In a community-based study of the relationship between bone lead and cardiac conduction among elderly men environmentally exposed to lead, our group previously found that higher bone lead levels were associated with electrocardiographic (ECG) features such as longer QT and QRS intervals and intra-ventricular and atrioventricular conduction defects, suggesting depressed cardiac conduction (Cheng et al. 1998). This could be evidence that even low-level exposure to lead may alter autonomic activity, because cardiac conduction is mediated, in part, through a branch of the autonomic nervous system (Ahnve and Vallin 1982).

Another issue in the study of lead toxicity is the identification of susceptible population groups, such as those with preexisting cardiovascular conditions. For example, people with metabolic syndrome (MetS), a cluster of health risks including obesity, diabetes, hypertension, and dyslipidemia (Moller and Kaufman 2005), are at greater risk of cardiovascular disease because they are likely to have stronger levels of oxidative-stress–induced inflammatory responses. As a result, they may be less able to homeostatically control responses to additional oxidative stressors such as lead exposure and therefore show more pronounced responses. Recently, Tsaih et al. (2004) observed that lead had a more pronounced effect on renal function in people with diabetes and hypertension. However, to our knowledge, no previous study has examined modifications of the association between lead exposure and HRV by clinical conditions, such as MetS, diabetes, and hypertension.

In this study we examined the association of low-level lead exposure (measured in bone) with alterations in HRV among community residents with no previously known heavy lead exposure. We also assessed effect modifications by MetS and its individual components.

## Materials and Methods

### Study population

The Normative Aging Study is a longitudinal study established by the Veterans Administration (VA; now the U.S. Department of Veterans Affairs) in 1963, when 2,280 community-dwelling men from the Greater Boston area 21–80 years of age were enrolled (Bell et al. 1972). All participants were free of known chronic medical conditions at enrollment. Every 3–5 years, participants underwent physical examination such as assessment of body mass index (BMI) and measurement of systolic and diastolic blood pressures. During these visits, participants filled out questionnaires on cigarette smoking, food frequency, and various health risk factors. In addition, data on fasting blood glucose, total cholesterol, and high-density lipoprotein (HDL) cholesterol were also obtained from blood samples. Participants visited the study center in the morning after an overnight fast and abstinence from smoking.

From 1991 to 2002, Normative Aging Study participants who gave their informed consent were invited to undergo bone lead measurements. If subjects had more than one bone lead measurement during this period (75% of subjects analyzed; mean = 1.7, maximum = 4), the measurement closest to the date of the HRV measurement was used for this analysis. In 98 subjects (23.7%), the bone lead levels were measured after the HRV measures were obtained. However, all of those bone lead measurements were obtained within 6 months of the HRV measurement. Because half-lives of tibia lead and patella lead are years to decades, we do not think that bone lead levels at the time of HRV measurement would have been different from those actually measured. In an earlier analysis, no important differences were detected between Normative Aging Study participants who did and did not have bone lead measurements taken (Cheng et al. 1998).

Beginning in November 2000, HRV measurement was added to the tests performed during the regular visits of Normative Aging Study subjects. Among active cohort members, 671 persons were examined for HRV from 14 November 2000 to 22 December 2004. Excluded were 110 subjects (16.4%) with problematic heart rate measurements, including atrial fibrillation, atrial bigeminy or trigeminy, pacemakers, irregular rhythm, irregular sinus rhythm, frequent ventricular ectopic activity, ventricular bigeminy, multi-focal atrial tachycardia, or measurement time < 3.5 min. We further excluded 131 and 142 subjects without tibia or patella lead measurements, respectively; 10 subjects with high bone lead measurement uncertainties (≥ 10 μg/g and 15 μg/g for tibia and patella, respectively); 5 and 9 subjects with extreme tibia and patella lead levels, respectively; and 2 subjects with missing values of the potential confounding factors. Hence, 413 (tibia) and 398 (patella) subjects were available for lead analyses. All participants had given written informed consent. This study was reviewed and approved by the institutional review boards of all participating institutions.

### HRV measurement

**Figure 1:**
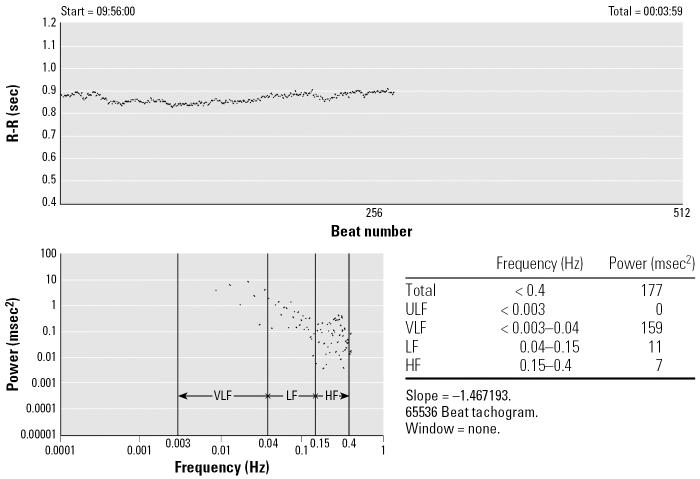
An example of time domain R-R interval data and the estimate of power spectral density. ULF, ultra-low frequency; VLF, very low frequency.

HRV was measured between 0600 and 1300 hours using a two-channel (five-lead) ECG monitor (Trillium 3000; Forest Medical, East Syracuse, NY). After the participants had rested for 5 min, the ECG was recorded (sampling rate of 256 Hz/channel) for approximately 7 min with the subject seated. We used the best 4-consecutive-min interval for the HRV calculations. The ECG digital recordings were processed, and heart rate and HRV measures were calculated using PC-based software (Trillium 3000 PC Companion Software for MS Windows; Forest Medical), which conforms to established guidelines (Task Force 1996). Beats were automatically detected and assigned tentative annotations, which were then reviewed by an experienced scanner to correct for any mislabeled beats or artifacts. We used only normal-to-normal (NN) beat intervals in the analysis; SD of NN intervals (SDNN) was calculated. We also computed high frequency (HF; 0.15–0.4 Hz), low frequency (LF; 0.04–0.15 Hz), and LF/HF ratio. HF and LF were also included as normalized units (HF_norm_ and LF_norm_), which reflect the relative value of each power component in proportion to the total power minus the very low frequency component. An example of time domain R–R interval data and the estimate of power spectral density is shown in Figure 1. Spectral analysis of the time domain transforms the signal from time to frequency on the *x*-axis using a Fast Fourier transformation, by representing the signal as a combination of sine and cosine waves, with different amplitudes and frequencies. Table 1 describes various HRV parameters measured in this study. HF represents an estimate of parasympathetic (vagal) activity. The interpretation of LF is more controversial. Some studies consider LF, when expressed in normalized units, to be a marker of sympathetic modulations, and other studies regard LF as reflecting both sympathetic and vagal activity. Consequently, LF/HF represents an estimate of sympathovagal balance or the sympathetic modulation (Task Force 1996). For simplicity, we present only the results for HF_norm_, LF_norm_, and LF/HF, which explain associations with the sympathetic and vagal modulations of the heart. Room temperature where the HRV measurement was taken was also recorded.

### Bone lead measurements

Bone lead levels were measured at the midtibial shaft and the patella using a K-shell X-ray fluorescence (KXRF) instrument (ABIOMED, Danvers, MA). The physical principles, technical specifications, and validation of this instrument have been described in detail (Burger et al. 1990). The tibia and patella were targeted for bone lead research because they consist mainly of pure cortical and pure trabecular bone, respectively, and thus represent the two main bone compartments. Lead in trabecular bone has a faster turnover rate and therefore reflects more recent exposure than that in cortical bone. The KXRF instrument provides an unbiased estimate of bone lead levels (normalized for bone mineral content as micrograms of lead per gram of bone mineral) and an estimate of the uncertainty associated with each measurement.

Most bone lead measurements were obtained before the HRV measurement (median, 3.2 years). Kim et al. (1997) reported that an individual’s patella bone lead decreased by 23% over a 3-year follow-up in this same population (7.67% decrease/year), but tibia lead levels did not change. To account for the declining trend in patella lead levels, we predicted estimated patella lead levels as


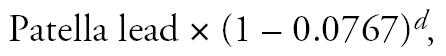


where *d* denotes the difference in years between dates of bone lead and HRV measurement.

### MetS and individual metabolic abnormalities

We used the criteria of the National Cholesterol Education Program Adult Treatment Panel III (Ford et al. 2002; Moller and Kaufman 2005) and defined subjects having three or more of the following criteria as having MetS: *a*) abdominal obesity (waist circumference > 102 cm in men); *b*) hyper-triglyceridemia (≥ 150 mg/dL); *c*) low HDL cholesterol (< 40 mg/dL in men); *d*) high blood pressure (≥ 130/85 mm Hg); and *e*) high fasting glucose (≥ 110 mg/dL). We counted subjects who reported currently using hypertension or diabetes medication as having high blood pressure or high fasting glucose, respectively. Subjects whose waist circumference measurement was missing (*n* = 13) were counted as having abdominal obesity if their BMI was ≥ 30 kg/m^2^.

To investigate effect modifications by individual components of the MetS, we used stricter definitions. Diabetes was defined as fasting blood glucose of ≥ 126 mg/dL, a physician’s diagnosis of type 2 diabetes, and/or use of a diabetes medication (e.g., oral hypoglycemic drug, metformin, or insulin). Hypertension was defined as reported use of hypertension medication, systolic blood pressure of ≥ 160 mmHg, or diastolic blood pressure of ≥ 96 mmHg. Because the prevalence of high blood pressure was relatively high in this population (67%), these high cutoff points were chosen to maintain a high degree of specificity among those subjects assigned as hypertensive (Hu et al. 1996). Abdominal obesity was defined as waist circumference ≥ 106 cm (75th percentile). Dyslipidemia was defined as hypertriglyceridemia (triglyceride of ≥ 150 mg/dL) and low HDL cholesterol levels (< 40 mg/dL).

### Statistical methods

Extreme outliers in bone lead measures were identified and removed using the generalized extreme studentized deviation many-outlier method (Rosner 1983), as in previous analyses (Hu et al. 1996).

Linear regression analyses were conducted to evaluate the relation of HRV with each lead marker. LF/HF was log_10_-transformed to improve normality and stabilize variance. The potential confounding factors were age, BMI, fasting blood glucose, HDL, triglyceride, cigarette smoking (current/former/never), alcohol consumption (two or more drinks a day, yes/no), use of beta-blockers, use of calcium channel blockers, use of angiotensin converting enzyme (ACE) inhibitors, room temperature, and season. We present effect estimates for an increment of the interquartile range (IQR) for each lead marker. For log-transformed LF/HF, the percent change for an increase of the IQR for each lead marker was estimated as [10^(β × IQR)^ − 1] × 100%, with 95% confidence intervals (CIs) {10^[IQR × (β ± 1.96 × SE)]^ − 1} × 100%, where β is the estimated regression coefficient. To assess modifying effects of MetS and other metabolic abnormalities, we ran regression models including multiplicative interaction terms along with the main effects.

To evaluate the linear regression assumption, penalized splines were used to allow the relation between the exposure and response to be more flexible (Eilers and Marx 1996). This smoothing method makes no assumptions regarding the shape of the association (Wood 2000). The penalized splines can be estimated in a generalized additive model using R software (R Foundation for Statistical Computing 2006). The optimal degree of smoothing was determined by the generalized cross-validation criterion, which is, in practice, an approximation of Akaike’s information criterion (Wood 2000).

## Results

Table 2 shows the demographic and clinical characteristics and HRV measurements of the participants. All the study participants were male, with a mean (± SD) age of 72.9 ± 6.5 years. The median tibia and patella lead levels were 19 μg/g (IQR, 11–28 μg/g) and 23 μg/g (IQR, 15–34 μg/g), respectively. After adjusting for the decreasing trend of patella lead levels, the estimated median was reduced (16.3 μg/g; IQR, 10.4–25.8 μg/g). Hereafter, we refer to the estimated value as “patella lead.” The correlation between tibia and patella lead was high (Spearman correlation coefficient = 0.54). Of the subjects, 133 (32%) were identified as having MetS. BMI, waist circumference, systolic blood pressure, fasting glucose, total cholesterol, HDL, and triglyceride showed statistically significant linear relations with the number of metabolic abnormalities. People with MetS (three or more metabolic abnormalities) were more likely to have ischemic heart disease and stroke and to be taking hypertension medications. In addition, subjects with MetS showed depressed HRV measures compared with subjects with two or fewer metabolic abnormalities, but these differences were not statistically significant.

Table 3 presents the prevalence of individual metabolic abnormalities and MetS and their relationship with tertiles of age-adjusted tibia and patella lead levels. The prevalence of high blood pressure and hypertriglyceridemia appeared to increase across tertiles of tibia lead levels, whereas those trends were not observed with patella lead levels. Instead, marginally significant reducing trends in the prevalence of abdominal obesity and MetS were found in relation to patella lead levels. No statistically significant difference was found in the prevalence of high fasting glucose in relation to both tibia and patella lead levels.

**Figure 2:**
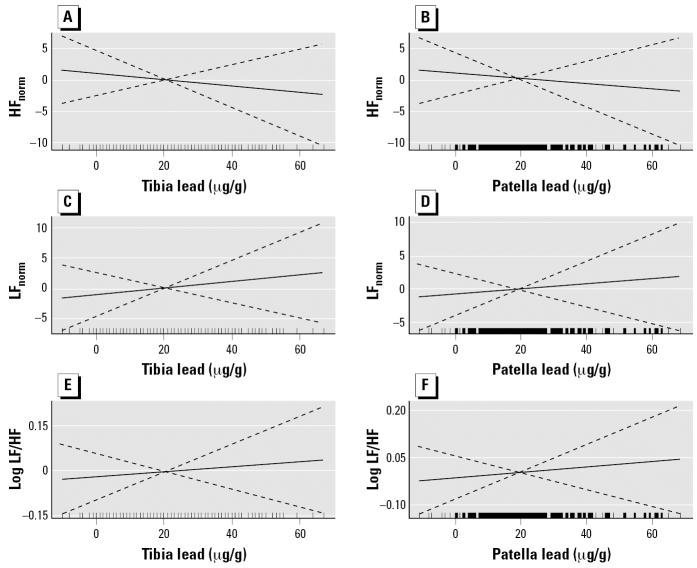
Associations of the HRV measures HF_norm_ (*A,B*), LF_norm_ (*C,D*) and log LF/HF (*E,F*) with tibia lead (*A,C,E*) and patella lead (*B,D,F*) adjusted for age, BMI, fasting glucose, HDL, triglyceride, cigarette smoking, alcohol consumption, use of β-blockers, calcium channel blockers, and/or ACE inhibitors, room temperature, and season. The solid line indicates the nonparametric trends estimated from the penalized spline method, and the dotted lines indicate its 95% CIs. The optimal degree of smoothing determined by the generalized cross-validation criterion for all HRV measures was 1, which means the associations were nearly linear.

Table 4 shows the estimated change and 95% CI of various HRV parameters per one IQR increase in each bone lead marker. After controlling for potential confounders, we found inverse but nonstatistically significant associations of both tibia and patella lead levels with HF_norm_, and nonstatistically significant positive relations with LF_norm_ and LF/HF. Smoothing analysis showed that those associations were nearly linear for all HRV markers (Figure 2). We examined whether the status of metabolic abnormalities modified the effects of bone lead on HRV (Table 4). For one IQR increase in patella lead (15.4 μg/g), HF_norm_ decreased by −3.9 nu (95% CI, −8.2 to 0.5 nu) and LF_norm_ increased by 3.9 nu (95% CI, −0.4 to 8.3 nu) among persons with MetS. We also observed a marginally significant positive association between patella lead and LF/HF among subjects with MetS (21.2% increase; 95% CI, −1.9 to 49.8%). However, we found no statistically significant association in people with two or fewer metabolic abnormalities. Furthermore, higher patella lead was associated with a graded significant reduction in HF_norm_ (*p* for trend = 0.048) and increases in LF_norm_ (*p* for trend = 0.047) and LF/HF (*p* for trend = 0.045) as the number of metabolic abnormalities increased. No statistically significant interaction was found between metabolic abnormalities and tibia lead in relation to HRV measures.

**Figure 3:**
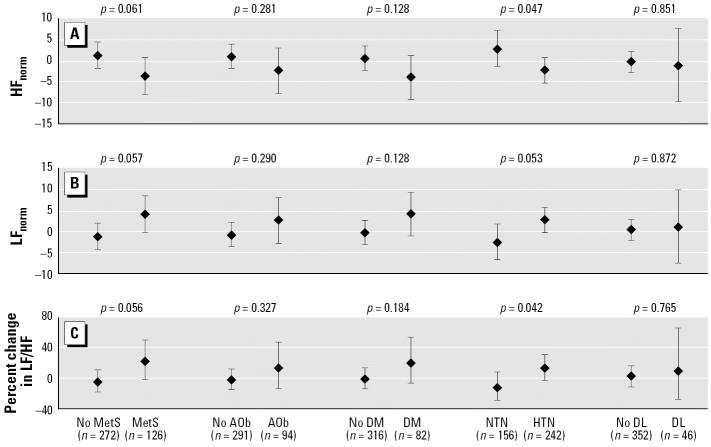
The estimated change and 95% CI in HRV measures associated with an IQR increase (15.4 μg/g) in patella lead levels by MetS, abdominal obesity (AOb), diabetes (DM), hypertension (HTN), and dyslipidemia (DL). The *p*-values above each component are for the interaction term. Interaction by MetS (model 1): adjusted for age, cigarette smoking, alcohol consumption, room temperature, and season; interaction by AOb: model 1 + fasting glucose, HDL, triglyceride, use of β-blockers, calcium channel blockers, and/or ACE inhibitors; interaction by DM: model 1 + BMI, HDL, triglyceride, use of β-blockers, calcium channel blockers, and/or ACE inhibitors; interaction by HTN: model 1 + BMI, HDL, triglyceride; interaction by DL: model 1 + BMI, fasting glucose, use of β-blockers, calcium channel blockers, and/or ACE inhibitors.

We also assessed effect modifications by individual components of MetS, such as abdominal obesity, diabetes, hypertension, and dyslipidemia. We only report results for patella lead (Figure 3) because no statistically significant interaction with tibia lead was found. When we combined data on persons with two or fewer metabolic abnormalities and compared them with data on persons with MetS, the differences in the effect of patella lead on all HRV measures were marginal. We did find statistically significant interactions between hypertension (use of antihypertensive medication or blood pressure ≥ 160/96 mmHg) and patella lead for HF_norm_ (*p* = 0.047 for interaction term) and LF/HF (*p* = 0.042 for interaction term). For each one IQR increase in patella lead, HF_norm_ decreased by 2.5 nu (95% CI, −5.5 to 0.55 nu) in persons with hypertension, whereas it increased by 2.7 nu (95% CI, −1.5 to 7.0 nu) in persons without hypertension. Similarly, among the same groups of subjects, one IQR increase in patella lead was associated with a 13.0% (95% CI, −2.4 to 30.8%) change in LF/HF among subjects with hypertension and a −12.9% (95% CI, −29.2 to 7.0%) change in LF/HF among those without hypertension. In addition, higher patella lead was consistently associated with lower HF_norm_ and higher LF_norm_ and LF/HF among obese or diabetic persons. However, the associations were not significant whether persons had those conditions or not, and interactions were not statistically significant. We also examined whether obesity, ischemic heart disease, use of statin, or use of antihypertensive medication, such as β-blockers and calcium channel blockers, modified the effects of bone lead on HRV, but no statistically significant interaction was observed (data not shown).

## Discussion

Results of the present study suggest that persons with multiple metabolic abnormalities are subject to significantly stronger effects from cumulative lead exposure, as reflected in patella bone, on a reduction in HF_norm_, a marker of parasympathetic (vagal) nerve activity, and on elevations in LF_norm_, a marker of sympathetic activity, and LF/HF ratio, a marker of sympathovagal balance. These associations became gradually stronger as the number of metabolic abnormalities increased. We also observed subjects with MetS or individual components were consistently more affected by patella lead than those without MetS or individual components. Elevated LF/HF reflects sympathetic excitation and parasympathetic withdrawal, which may be linked with cardiac events such as ventricular arrhythmias and myocardial infarction (Task Force 1996). When the cohort was not stratified by number of metabolic abnormalities, trends were in the expected direction but were not statistically significant, presumably because of the direction of the association among people without metabolic abnormalities being opposite to the association among people with MetS.

The biologic mechanisms that link lead exposure with alterations in the autonomic nervous system are not well understood. Redox-inactive metals such as lead deplete glutathione and protein-bound sulfhydryl groups, resulting in the production of ROS such as hydroxyl radicals, because lead has a high affinity for sulfhydryl groups (Gurer and Ercal 2000). Many studies have confirmed that lead exposure is associated with lipid peroxidation, alterations in antioxidant enzyme activities such as superoxide dismutase and glutathione peroxidase, and DNA damage (Fracasso et al. 2002; Oktem et al. 2004; Sandhir and Gill 1995; Ye et al. 1999). Oxidative stress is known to produce proinflammatory mediators, inhibit nitric oxide, and alter calcium homeostasis, which may be linked with sympathetic excitation and vagal withdrawal (Ding et al. 2000; Dursun et al. 2005; Vaziri 2002). Lead modifies the intracellular calcium messenger system and disturbs calcium homeostasis because lead mimics calcium and enters cells via calcium channels (Kober and Cooper 1976; Sandhir and Gill 1994). Lead exposure down-regulates nitric oxide production (Vaziri 2002), which causes an increase in sympathetic and a reduction in vagal activity (Chowdhary et al. 2002).

Conflicting results were reported in studies of workers occupationally exposed to lead. Studies of male workers with above-average blood lead levels (average ~ 35 μg/dL) found significantly diminished vagal activity as measured by the coefficient of variation in R–R intervals compared with nonexposed controls at rest (Murata and Araki 1991; Murata et al. 1993) and during deep breathing (Teruya et al. 1991), whereas no association was found among battery workers with mean blood lead of 51 μg/dL (Gennart et al. 1992). These conflicting results may be due to different geographical areas and age groups studied, or because investigators did not consider potential effect modification by the underlying oxidative stress, such as metabolic syndrome.

MetS, also known as syndrome X or insulin resistance syndrome, is a set of cardiovascular risks that increases the likelihood of developing type 2 diabetes, hypertension, and coronary artery disease (Moller and Kaufman 2005). From analysis based on the National Health and Nutrition Examination Survey III data, the age-adjusted prevalence of MetS was 24% overall and 42% among those ≥ 70 years of age (Ford et al. 2002). An estimated 47 million U.S. residents have MetS based on 2000 census data (Ford et al. 2002). Several large prospective studies have shown associations between MetS and increased risks of cardiovascular disease as well as all-cause mortality (Ford 2005; Malik et al. 2004). In the present study, approximately 32% of the participants (mean age 73 years) had MetS; this relatively low prevalence probably reflects the predominant white population of the Normative Aging Study cohort.

MetS is known to be associated with low HRV (Hemingway et al. 2005; Liao et al. 1998; Pikkujamsa et al. 1998). This is likely because people with MetS tend to have a greater percentage of adipose tissue (related to insulin resistance, glucose intolerance, and increased inflammation), atherogenic dyslipidemia, hypertension, and a higher proinflammatory and prothrombotic state, all of which are associated with decreased parasympathetic and increased sympathetic tone (Moller and Kaufman 2005). A possible explanation of our results is that the combined oxidative stress and resulting overexposure to ROS caused by long-term exposure to lead and MetS increases the risk of autonomic dysfunction. The observed significant interaction between hypertension and patella lead levels in relation to HRV may have a similar explanation: hypertension is associated with depressed HRV (Schroeder et al. 2003; Singh et al. 1998) and has been shown to modify the association between HRV and other ROS-generating factors, such as particulate matter (Park et al. 2005). Nonstatistically significant but consistent alterations in HRV measures only among persons with diabetes or abdominal obesity support the contention that proinflammatory conditions play a role in susceptibility to the autonomic impact of lead exposure.

We found that patella lead, but not tibia lead, had interactive effects with MetS on HRV. This differential sensitivity may be due to different lead kinetics in the two types of bone, cortical (tibia) bone and trabecular (patella) bone. Because the half-life of lead is decades in the tibia and only a few years in the patella, tibia lead reflects long-term cumulative lead exposure, whereas patella lead indicates the predominant skeletal source of circulating lead (Hu et al. 1998). Because patella lead is a more readily mobilizable bone source of lead than tibia lead, it may be a better marker in the association with HRV.

We could not evaluate the association between blood lead and HRV because most blood lead measurements among the Normative Aging Study participants were not obtained at the time of HRV measurement; thus, those levels could not be presumed to be a marker of lead exposure at the time of HRV measurement. As discussed above, several studies have shown an association between blood lead and alterations in autonomic function among occupationally exposed subjects with very high blood lead levels (Bockelmann et al. 2002; Murata and Araki 1991; Murata et al. 1993; Teruya et al. 1991). Therefore, further studies of the relationship between blood lead and HRV in a general population not occupationally exposed to lead are needed to determine whether contemporary exposure to lead is an important predictor of autonomic dysfunction.

The present study has several limitations. Although we collected information on many factors that might influence autonomic nervous system function, those factors could not account for all the variation in HRV. However, we tried to control for many potential confounding factors including age, BMI, blood glucose, cigarette smoking, and alcohol consumption. We controlled for blood lipid profiles (HDL and triglyceride), although these did not change the associations we observed because these variables account for some variance in HRV. We measured the ECG at a stable temperature and adjusted for the temperature of the room where the ECG was taken. Additionally, we adjusted for season, because HRV indexes of healthy men vary physiologically by season, with lowest values in the winter (Kristal-Boneh et al. 2000). Therefore, the observed findings are less likely to reflect bias due to these confounding factors.

We analyzed subjects who had bone lead levels available (approximately 77% of all Normative Aging Study subjects who had ECG measurements). However, our research group has previously found no important differences between Normative Aging Study participants who did and did not have bone lead levels available (Cheng et al. 1998). Therefore, we believe that the participants of this study represent the entire cohort.

In addition, the Normative Aging Study cohort is all male and almost all white. Thus, while sex and race cannot bias our results (both are known to be important determinants of HRV), the results of this study may not be generalizable to females or nonwhite ethnic populations.

This population-based study supports the hypotheses that the autonomic effects of cumulative lead exposure become gradually stronger with the number of metabolic abnormalities and that elderly men with individual components of MetS are more susceptible to cardiac autonomic dysfunction (as measured by HRV) in association with chronic lead exposure as measured in patella. The modification by MetS could be consistent with the hypothesis that the oxidative stress response is an important mechanism by which lead toxicity affects the cardiovascular system.

## Figures and Tables

**Table 1:** HRV measures used in this study.

HRV measures (units)	Description
SDNN (msec)	SD of all NN intervals, an estimate of overall variability
HF (msec^2^)	High frequency power (0.15–0.4 Hz), a marker of parasympathetic (vagal) modulation
HF_norm_ (nu)	HF power in normalized units, HF/(total power – very low frequency power) × 100
LF (ms^2^)	Low frequency power (0.04–0.15 Hz), a marker of both sympathetic and vagal modulations
LF_norm_ (nu)	LF power in normalized units, LF/(total power – very low frequency power) × 100, a marker of sympathetic modulation
LF/HF	Ratio LF [ms^2^]/HF[ms^2^], sympathovagal balance OR the sympathetic modulation

**Table 2:** Characteristics of study population according to the number of metabolic abnormalities.

	Study participants			
		No. of metabolic abnormalities^a^		
	All (*n* = 413)	0 (*n* = 60)	1–2 (*n* = 220)	≥ 3 (*n* = 133)
Bone lead [median (IQR)]				
Tibia lead (μg/g)	19.0 (11.0–28.0)	18.5 (10.5–23.0)	19.0 (11.0–28.0)	19.0 (12.0–26.0)
Patella lead (μg/g)^b^	23.0 (15.0–34.0)	22.0 (13.5–32.0)	25.0 (16.0–36.0)	20.0 (15.0–32.0)
Estimated patella lead (μg/g)^b^,^c^	16.3 (10.4–25.8)	16.3 (10.8–24.8)	17.1 (11.0–29.3)	15.1 (9.4–22.1)
Continuous variables (mean ± SD)				
Age (years)	72.9 ± 6.5	72.4 ± 7.7	73.5 ± 6.3	72.3 ± 6.0
Body mass index (kg/m^2^)	28.0 ± 4.0	25.0 ± 2.6	27.0 ± 3.0	30.9 ± 4.1*
Waist circumference (cm)^d^	99.7 ± 9.8	91.2 ± 6.6	97.5 ± 7.5	107.3 ± 9.3*
Systolic blood pressure (mmHg)	130.6 ± 16.8	125.3 ± 14.5	131.0 ± 17.1	132.3 ± 17.0*
Diastolic blood pressure (mmHg)	74.6 ± 10.1	73.5 ± 8.0	74.6 ± 10.2	75.0 ± 10.8
Heart rate (beats/min)	70.8 ± 6.9	70.9 ± 5.5	70.6 ± 6.7	71.0 ± 7.8
Fasting blood glucose (mg/dL)	107.6 ± 27.8	94.3 ± 7.3	101.1 ± 16.3	124.3 ± 39.0*
Cholesterol (mg/dL)	192.8 ± 37.0	205.9 ± 30.5	192.9 ± 36.6	186.7 ± 39.5*
High density lipoprotein (mg/dL)	49.3 ± 13.6	60.3 ± 16.0	51.2 ± 12.0	41.3 ± 9.8*
Triglyceride (mg/dL)	126.5 ± 67.6	86.5 ± 30.1	105.4 ± 47.2	179.4 ± 76.6*
Categorical variables (%)				
Smoking status				
Never-smoker	31.2	38.3	30.9	28.6
Former smoker	63.2	55.0	64.1	65.4
Current smoker	5.6	6.7	5.0	6.0
Alcohol intake (≥ 2 drinks/day)	19.4	18.3	22.7	14.3
Diabetes mellitus	20.6	0.0	10.9	45.9*
Ischemic heart disease history	29.1	13.3	29.6	35.3*
Stroke history	6.3	0.0	6.8	8.3*
Hypertension	70.9	11.7	76.8	88.0*
Use of β-blocker	34.6	0.0	36.4	47.4*
Use of Ca-channel blocker	14.0	0.0	14.1	20.3*
Use of ACE inhibitor	21.3	0.0	22.3	29.3*
Use of statin	37.1	20.0	35.0	48.1*
HRV [mean (median)]				
SDNN (msec)	38.8 (34.0)	39.8 (35.5)	40.7 (35.0)	35.2 (30.0)
HF (msec^2^)	290.1 (71.0)	268.0 (79.0)	346.1 (67.5)	207.4 (71.0)
LF (msec^2^)	193.5 (96.0)	194.0 (117.5)	213.8 (100.0)	159.7 (83.0)
HF_norm_ (nu)	45.8 (41.8)	44.0 (40.1)	45.0 (39.7)	48.0 (47.4)
LF_norm_ (nu)	54.2 (58.2)	56.1 (59.9)	55.0 (60.3)	51.9 (52.6)
LF/HF	2.1 (1.4)	2.2 (1.5)	2.2 (1.5)	1.8 (1.1)

a Abdominal obesity, hypertriglyceridemia, low HDL cholesterol, high blood pressure, and high fasting glucose.

b *n* = 398.

c Patella lead × (1 − 0.0767)^(difference in years between dates of bone lead and HRV measurement)^.

d *n* = 400.

* *p* for trend < 0.05.

**Table 3:** Prevalence of individual metabolic abnormalities and MetS in the Normative Aging Study.

	Abdominal obesity	Hypertriglyceridemia	Low HDL cholesterol	High blood pressure or medication use	High fasting glucose or medication use	MetS
Total (%)^a^	35.8	26.4	25.4	66.8	33.4	32.2
Tertile of age-adjusted tibia lead (%)						
< 14.1 μg/g (*n* = 138)	36.2	22.5	23.2	64.5	33.3	31.2
14.1–23.8 μg/g (*n* = 138)	37.2	25.4	24.6	60.9	29.7	30.4
> 23.8 μg/g (*n* = 137)	35.0	31.4	28.5	75.2	37.2	35.0
*p* for trend	0.84	0.09	0.32	0.06	0.50	0.49
Tertile of age-adjusted patella lead^b^ (%)						
< 13.2 μg/g (*n* = 132)	40.2	28.8	31.8	65.9	31.1	37.1
13.2–22.2 μg/g (*n* = 133)	36.8	29.3	24.1	63.9	35.3	33.1
> 22.2 μg/g (*n* = 133)	29.3	21.1	23.3	66.9	34.6	27.1
*p* for trend	0.07	0.15	0.12	0.86	0.54	0.08

a *n* = 413.

b *n* = 398.

**Table 4:** The estimated change (95% CI) in HRV parameters associated with one IQR increase in bone lead markers.

	Tibia				Patella			
	No.	HF_norm_ (nu)	LF_norm_ (nu)	Log_10_ LF/HF (%)	No.	HF_norm_ (nu)	LF_norm_ (nu)	Log_10_ LF/HF (%)
All								
Model 1	413	−1.1 (−4.1 to 1.9)	1.2 (−1.8 to 4.2)	4.6 (−9.8 to 21.3)	398	−0.6 (−3.1 to 1.9)	0.6 (−1.9 to 3.1)	3.0 (−8.9 to 16.5)
Model 2	413	−0.9 (−3.8 to 2.1)	0.9 (−2.0 to 3.9)	3.3 (−10.7 to 19.5)	398	−0.6 (−3.1 to 1.9)	0.6 (−1.9 to 3.1)	3.0 (−8.7 to 16.2)
No. of metabolic abnormalities^a^								
0	60	−3.8 (−12.5 to 4.9)	4.0 (−4.8 to 12.7)	22.6 (−20.3 to 88.4)	60	3.7 (−3.8 to 11.2)	−3.5 (−11.0 to 4.0)	−16.1 (−41.7 to 20.8)
1–2	220	0.1 (−3.7 to 3.9)	−0.03 (−3.9 to 3.8)	−3.0 (−19.7 to 17.2)	209	0.7 (−2.6 to 4.0)	−0.8 (−4.1 to 2.5)	−3.4 (−17.8 to 13.4)
≥ 3	133	−2.7 (−7.9 to 2.5)	2.7 (−2.5 to 7.9)	15.6 (−10.5 to 49.4)	129	−3.9 (−8.2 to 0.5)*	3.9 (−0.4 to 8.3)*	21.2 (−1.9 to 49.8)*
*p-*Value for trend		0.896	0.920	0.808		0.048	0.047	0.045

IQR increase: 17 μg/g for tibia lead and 15.4 μg/g for patella lead. Model 1: adjusted for age, cigarette smoking, alcohol consumption, room temperature, and season. Model 2: further adjusted for BMI; fasting glucose; HDL cholesterol; triglyceride; and use of β-blockers, calcium channel blockers, and/or ACE inhibitors.

a All models adjusted as model 1.

* *p* < 0.1.
